# Interaction of certain monoterpenoid hydrocarbons with the receptor binding domain of 2019 novel coronavirus (2019-nCoV), transmembrane serine protease 2 (TMPRSS2), cathepsin B, and cathepsin L (CatB/L) and their pharmacokinetic properties

**DOI:** 10.3906/biy-2005-46

**Published:** 2020-06-21

**Authors:** Erman Salih İSTİFLİ, Arzuhan ŞIHOĞLU TEPE, Cengiz SARIKÜRKCÜ, Bektaş TEPE

**Affiliations:** 1 Department of Biology, Faculty of Science and Literature, Çukurova University, Adana Turkey; 2 Department of Biology, Faculty of Science and Literature, Gaziantep University, Gaziantep Turkey; 3 Department of Analytical Chemistry, Faculty of Pharmacy, Afyonkarahisar Health Sciences University, Afyonkarahisar Turkey; 4 Department of Molecular Biology and Genetics, Faculty of Science and Literature, Kilis 7 Aralık University, Kilis Turkey

**Keywords:** 2019-nCoV, ACE2, TMPRSS2, cathepsin, monoterpene, docking, pharmacokinetic

## Abstract

As of June 2020, the coronavirus disease 19 (COVID-19) caused by the 2019 new type coronavirus (2019-nCoV) infected more than 7,000,000 people worldwide and caused the death of more than 400,000 people. The aim of this study was to investigate the molecular interactions between monoterpenoids and spike protein of 2019-nCoV together with the cellular proteases [transmembrane serine protease 2 (TMPRSS2), cathepsin B (CatB), and cathepsin L (CatL)]. As a result of the relative binding capacity index (RBCI) analysis, carvone was found to be the most effective molecule against all targets when binding energy and predicted (theoretical) IC50 data were evaluated together. It was found to exhibit drug-likeness property according to the Lipinski’s rule-of-five. Carvone has also been determined to be able to cross the blood-brain barrier (BBB) effectively, not a substrate for P-glycoprotein (P-gp), not to inhibit any of the cytochrome P molecules, and to have no toxic effects even on liver cells. In addition, the LD50 dose of carvone in rats was 1.707 mol/kg. Due to its interaction profile with target proteins and excellent pharmacokinetic properties, it has been concluded that carvone can be considered as an alternative agent in drug development studies against 2019-nCoV.

## 1. Introduction

Some Coronaviridae viruses are in circulation among people and are known to cause mild respiratory infections (Corman et al., 2019). However, it has been established that 2 important members of this family are transmitted from animals to humans and cause serious infections. These are severe acute respiratory syndrome coronavirus (SARS-CoV) and Middle East respiratory syndrome coronavirus (MERS-CoV). Both types have led to severe respiratory infections in humans and the death of some individuals, especially those with chronic conditions (Fehr et al., 2017). SARS was first seen in Guangdong, China in 2002 and then quickly spread to other countries. This variant caused 8,096 people to become infected and 774 of these individuals died^1^1WHO (2003). Summary of probable SARS cases with onset of illness from 1 November 2002 to 31 July 2003 [online]. Website https://wwwwhoint/ csr/sars/country/table2004_04_21/en/ [accessed 00 Month Year]. (De Wit et al., 2016). The main source of SARS-CoV has been found to be Chinese horseshoe bats (Lau et al., 2005; Li et al., 2005). It has been reported to be transferred to humans through civet cats and raccoon dogs sold as food at Chinese wet markets (Guan et al., 2003). There is no approved antiviral agent or vaccine used in the treatment of SARS, either at the time of its first appearance or now. The spread of the pandemic that emerged in the period of 2002–2003 has been prevented by using traditional methods such as restricting people’s travel and isolation of sick individuals, just like today (Hoffmann et al., 2020).

After the epidemics of SARS and MERS in the early millennium, a new and highly contagious respiratory disease was detected in Wuhan (China) towards the end of 2019 (Huang et al., 2020; Wang et al., 2020; Zhu et al., 2020). It was reported that the first infected were people who came into contact with animals in the Huanan seafood market. Later it became clear that the virus could spread among humans (Chan et al., 2020). The so-called coronavirus disease 19 (COVID-19) spread rapidly to all parts of China. A new variant of SARS virus was found in the analysis of samples taken from sick individuals. The pathogen that caused the disease was called SARS-coronavirus 2 (SARS-CoV-2) or 2019 new coronavirus (2019-nCoV) because it is from the same family as SARS-CoV (Zhu et al., 2020). In February 2020, nearly 45,000 cases were detected in China. It has been announced that about 8,000 of these cases are in critical condition and over 1,000 people have died^2^2WHO (2020). Novel Coronavirus (2019-nCoV): situation report, 3 [online]. Website https://www.who.int/docs/default-source/coronaviruse/ situation-reports/20200212-sitrep-20200223-ncov.pdf?sfv rsn=20200241e20200219fb20200278_20200214 [accessed 08 May 2020].. The virus has spread to about 2 dozen countries, primarily through people traveling from China to other countries. As of June 2020, the number of COVID-19 cases worldwide has exceeded 7,000,000. More than 400,000 of these patients died as of early June 2020^3^3Worldometer (2020). COVID-19 coronavirus pandemic [online]. Website https://www.worldometers.info/coronavirus/ [accessed 08 May 2020]. . At present, it is not possible to make any inference about the sequence similarities between the SARS-CoV-2 and the SARS-CoV on the pandemic properties of these 2 variants (Munster et al., 2020).

In both coronavirus variants, spike protein plays an important role in the entry of the pathogen into the cell. Entry occurs as a result of the interaction between the S1 subunit of the spike protein and the receptor on the surface of the target cell. However, for entry, priming of the spike protein by cellular proteases is required. In this process, the spike protein is cut at the S1/S2 point and the S2’unit provides the junction between the virus and the cellular membrane. The virus needs angiotensin-converting enzyme 2 (ACE2) to bind to the receptor on the target cell surface (Li et al., 2003). Research has shown that transmembrane serine protease 2 (TMPRSS2) is involved in the priming of spike proteins of SARS viruses (Matsuyama et al., 2010; Glowacka et al., 2011; Shulla et al., 2011). Since TMPRSS2 is actively involved in the priming process of the 2019-nCoV spike protein, there is some evidence that camostat mesylate, which has an inhibitory effect on this protease, prevents infection in lung cells. Cathepsin B and cathepsin L (CatB/L), which are endosomal cysteine proteases, are also thought to be involved in priming of the spike protein of 2019-nCoV. Thus, it has been shown that inhibition of these proteases may also prevent the virus from entering the cell (Hoffmann et al., 2020).

Plant secondary metabolites are synthesized by many species such as vegetables, fruits, medicinal and aromatic plants (Prakash et al., 2007; Singh et al., 2009; Singh et al., 2009; Singh et al., 2009; Singh et al., 2010). Some phytochemicals have been reported to have significant antiviral activity. Thus, great attention has been paid to plant secondary metabolites in the treatment of some viral infections. Various studies have been conducted on the potential of some of these phytochemicals to inhibit the receptor binding domain (RBD) of the spike protein of 2019-nCoV, cellular proteases or endoribonucleases (Meneguzzo et al., 2020; Sampangi-Ramaiah et al., 2020; Tallei et al., 2020; Thuy et al., 2020).

**Figure 1:**
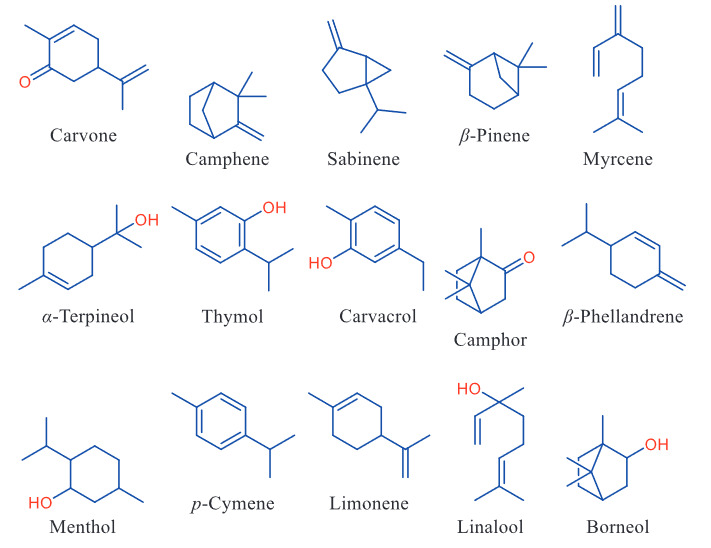
Chemical structures of the monoterpenoids.

The aim of this study was to investigate the molecular interactions between monoterpenoid hydrocarbons (Figure 1 and Table 1), which constitute an important group of plant essential oils, and i) RBD of the spike protein of 2019-nCoV and ii) cellular proteases (TMPRSS2, CatB and CatL). In addition, drug-likeness properties and ADMET profiles of monoterpenoids were presented. Using the binding free energy (kcal/mol) and predicted (theoretical) IC50 (mM) values of the monoterpenoids, the relative binding capacity index (RBCI) values were also statistically calculated and ‘hit’ compounds were determined.

**Table 1:** PubChem CID, molecular weight and molecular formula of the compounds.

No	Compound	PubChem CID	Molecular weight (g/mol)	Molecular formula
1	Carvone	7439	150.22	C10H14O
2	Camphene	6616	136.23	C10H16
3	Sabinene	18818	136.23	C10H16
4	β-Pinene	14896	136.23	C10H16
5	Myrcene	31253	136.23	C10H16
6	α-Terpineol	17100	154.25	C10H18O
7	Thymol	6989	150.22	C10H14O
8	Carvacrol	10364	150.22	C10H14O
9	Camphor	2537	152.23	C10H16O
10	β-Phellandrene	11142	136.23	C10H16
11	Menthol	1254	156.26	C10H20O
12	p-Cymene	7463	134.22	C10H14
13	Limonene	22311	136.23	C10H16
14	Linalool	6549	154.25	C10H18O
15	Borneol	64685	154.25	C10H18O

## 2. Materials and methods

### 2.1. Computational capacity 

Two Dell laptops (Windows 8.1 and Windows 10 operating system) were used to perform the molecular docking analyses in the current study. One of the computers had the Windows 10 operating system and was equipped with the Intel Core i5-7200U CPU 2.50 GHz and 2.70 GHz processors. The other computer with the Windows 8 operating system had Intel Core i5-5200U CPU 2.20 GHz and 2.20 GHz processor power.

### 2.2. Structural optimization of ligands

The protein data bank (.pdb) files of all the ligands have been downloaded from PubChem (https://pubchem.ncbi.nlm.nih.gov) via the download module of Vega ZZ 3.2.0.9 software. In the Vega ZZ, the atom types and electrical charges of the ligands were fixed with MMFF94 force field and Gasteiger-Marsili parameters (Pedretti et al., 2004). The ligands were energetically minimized by the conjugate gradient minimization method. For this purpose, the minimization steps and tolerance were set to 1000 and 0.01, respectively. 

### 2.3. Energy minimization of 2019-nCoV ACE2-RBD, TMPRSS2, CatB/L using nanoscale molecular dynamics (NAMD)

Firstly, in the Vega ZZ environment, the structure of the spike glycoprotein was gained by removing the ACE2 subunit from the angiotensin-converting enzyme 2 - 2019-nCoV receptor binding domain (RBD) complex which was downloaded from the url: https://swissmodel.expasy.org/interactive/HLkhkP/models/03 (PDB ID: model_03.pdb) (Camacho et al., 2009; Remmert et al., 2011). Since the structure of the spike glycoprotein in model_03 shows a sequence identity of 99.88% to the 2019-nCoV ACE2-binding domain, this model was chosen as the appropriate 3D structure in the docking analyses. During the protein preparation step, the atom types and electrical charges of the spike glycoprotein were fixed using CHARMM22_PROT force field and Gasteiger-Marsili charges. Next, for the energy minimization of the spike glycoprotein with NAMD, each parameter was loaded from a template file. The number of time steps (number of minimization steps) were set to 10,000 and CHARMM22_PROT was set as the force field. When the energy minimization was completed, the 3D structure corresponding to the last minimization step was saved as the lowest energy conformation. Also, to keep the spike glycoprotein structurally closer to the original crystallographic data, atom constraints were also applied to the protein backbone. In the energy minimization of TMPRSS2, CatB, and CatL, the same steps described for the 2019-nCoV spike glycoprotein were applied.

### 2.4. Homology modeling of TMPRSS2

Since the crystallographic data of TMPRSS2 enzyme structure has not been resolved until today, we generated a homology model to use in docking analyses with this enzyme. The amino acid sequence of TMPRSS2 was downloaded from UniProtKB (https://www.uniprot.org/uniprot/O15393)). Template search for TMPRSS2 catalytic domain was performed against the SWISS-MODEL template library with BLAST and HHBlits. BLAST was used to search the TMPRSS2 catalytic domain target sequence against the primary amino acid sequence in the SMTL. As a result of the BLAST search, a total of 788 templates were found. An initial HHblits profile has been built using the procedure as described in (Remmert et al., 2011)**.** This procedure was followed by 1 iteration of HHblits against NR20. The obtained profile was then searched against all profiles of the SMTL and, finally, a total of 1167 templates were found.

ProMod3 was used to carry out model building for TMPRSS2 catalytic domain based on the target-template alignment. The coordinates preserved between the target structure and the template were copied from the template to the model. Insertion and deletions were remodeled based on the fragment library. Subsequently, the side chains were rebuilt. Finally, using the CHARMM27 force field, the geometry of the resulting TMPRSS2 model was optimized. In cases where ProMod3 failed in loop modeling, an alternative model was developed with PROMOD-II (Guex et al., 2009).

The model quality (global and per-residue) of TMPRSS2 obtained was evaluated with the QMEAN scoring function (Studer et al., 2020). The near-zero QMEAN score reflects a good agreement between the model structure and the experimental structure, although scores of –4.0 and below indicate that the model is of low quality. Therefore, among the top 5 TMPRSS2 templates obtained as a result of homology modeling, the 5ce1.1.A (model 06) template with the QMEAN score closest to zero (QMEAN = –1.43) was selected as the target in the docking analysis.

In addition, whether our model has an energetically favorable conformation was analyzed by generating a Ramachandran plot in the PROCHECK (Laskowski et al., 1993) web-based tool. ERRAT (Colovos and Yeates 1993) online web-based tool was also deployed to calculate the overall quality factor (OQF) for nonbonded atomic interactions.

### 2.5. Molecular docking analyses

Molecular docking analyses was performed using AutoDock 4.2 to predict the binding affinities of carvone, camphene, sabinene, beta-pinene, myrcene, alpha-terpineol, thymol, carvacrol, camphor, β-phellandrene, menthol, *p*-cymene, limonene, linalool, borneol with 2019-nCoV RBD (PDB ID: model_03, https://swissmodel.expasy.org/interactive/HLkhkP/models/03),TMPRSS2 (model_06,https://swissmodel.expasy.org/interactive/HMKd4q/models/)), cathepsin B (PDB ID: 1GMY), and cathepsin L (PDB ID: 2YJ9). AutoDockTools-1.5.6 was used to prepare the target and ligand molecules and also the parameters prior to initiating the docking analysis using AutoDock 4.2 (Sanner, 1999). In this study, the grid box coordinates used in molecular docking analyzes were adjusted to ensure that all the tested phytochemicals interact with amino acids in the active sites of the enzymes in question (Greenspan et al., 2001; Wilson et al., 2005; Hardegger et al., 2011; Andersen et al., 2020).

In the molecular docking analyzes, polar hydrogen atoms in the receptor and the ligand molecules were retained while nonpolar hydrogens were merged and then, the Gasteiger charges of the ligands were calculated with AutoDockTools as previously described (Ricci and Netz 2009; Nasab et al., 2017). In addition, the Kollmann charges were added for the receptor. During the docking experiments, all the rotatable bonds of the ligands were allowed to rotate and then the optimized protein (rigid) and ligand (flexible) structures were saved in PDBQT format. Grid box coordinates were adjusted as: a) 80 × 90 × 40 Å points for the spike glycoprotein; b) 60 × 110 × 86 Å points for TMPRSS2; c) 86 × 84 × 44 Å points for CatB; and d) 54 × 52 × 60 Å points for CatL. Prior to docking analyses, these grid box sizes were determined to include the active amino acid residues of these enzymes.

In all docking analyses, 50 genetic algorithm (GA) runs using an initial population of 150 individuals, maximum number of 2,500,000 energy evaluations, and a maximum number of 27,000 generations were selected. The values of 0.02 and 0.8 were chosen as the default parameters for mutation and crossover rates, respectively. After 50 independent docking runs, all the possible binding modes (conformations) of the ligands were clustered by the program and were ranked based on the lowest RMSD (root mean square deviation) and the binding free energy (kcal/mol) of the ligand conformation. The best docking poses obtained using the AutoDock 4.2 between the ligand and receptor structures was analyzed with the BIOVIA Discovery Studio Visualizer 2016.

### 2.6. Success criteria set in docking analysis

In the current study, the RMSD (root mean square deviation) value of the docking results obtained for each phytochemical analyzed was considered successful when it was less than 2 angstroms (<2 Å). The criterion considered after the RMSD value was the binding energy (deltaG) of the ligand in the most efficient docked complex. Briefly, the closeness of all the phytochemicals tested in this study to the ACE2-RBD, TMPRSS2, CatB and CatL, and then the energy of that binding in these zones were determined (Morris and Lim-Wilby, 2008). The calculated inhibition constants (Ki) obtained with AutoDock 4.2 for each docked phytochemical were also given. 

### 2.7. Drug-likeness prediction, ADMET profile and target prediction

The drug-likeness, ADMET and target profiles of potential hit compounds are very important in terms of reducing side effects in the pharmaceutical industry. In our study, web-based SwissADME, pkCSM and Swiss Target Prediction online tools were used to determine such effects of monoterpene hydrocarbons analyzed (Pires et al., 2015; Daina et al., 2017; Daina et al., 2019).

### 2.8. Calculation of RBCI values

A new analysis method called RBCI was applied to statistically rank the activity potentials of phytochemicals by using binding energy and IC50 values obtained from the parameters given above. Through this analysis, it is possible to compare data, each of which has different scientific meanings, statistically with each other. If sorting based on the interaction of molecules with proteins is performed according to only 1 of these parameters (eg binding energy or IC50 value only), the molecules can only be sorted in terms of their potential in that parameter. However, sorting using only 1 of these parameters cannot represent the activity potential of these molecules from all parameters.

The most commonly used method to determine the interaction between the receptor-ligand in multiple measurements is the central tendency, in which the components are ranked-based on the mean value for each component (Zar, 1996). However, since the units and scales of the data obtained from each parameter are different, it is not possible to obtain an average value for all components.If the values in each data set (binding energy and IC50) are converted to standard scores, it is possible to compare them with each other.

In order to calculate the arithmetic mean values, first of all, binding energy and IC50 data of each phytochemical were used regardless of their units and raw values were obtained. These raw values calculated for each component were subtracted from the arithmetic mean and divided by standard deviation, and standard scores were obtained (see equation given below) (Sharma, 1996)). RBCI values of each phytochemical was calculated by averaging these standard scores obtained separately for each protein target.

where ‘x’ is the raw data, ‘μ’ is the mean, and ‘σ’ is the standard deviation.

Although RBCI is a relative index and does not represent the specific binding capacities of the components, it makes it possible to rank components reasonably based on their binding energy and IC50 values. Therefore, it can be used as an integrated approach to evaluate the molecular interaction of the components, considering all parameters.

## 3. Results

### 3.1. Homology modeling

**Figure 2:**
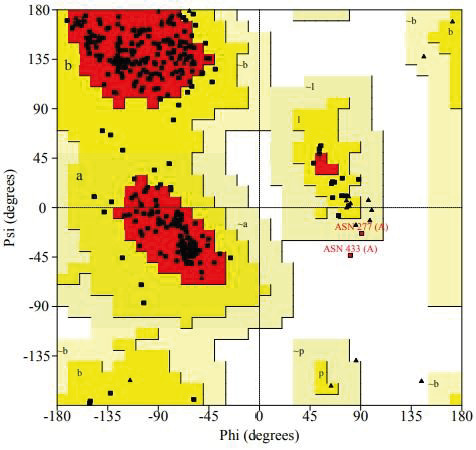
Ramachandran plot of TMPRSS2 model.

**Figure 3:**
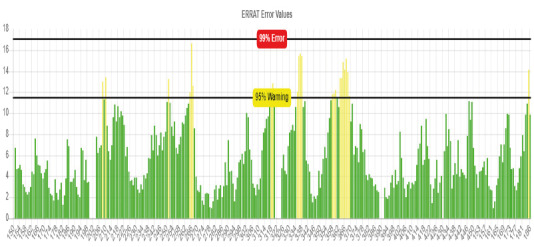
ERRAT error values for TMPRSS2 model. (Protein regions show misfolding at 95% confidence level are indicated with yellow bars. Green bars, on the other hand, point to regions that show correct folding.)

Of the 5 models created by ProMod3, model 06, which had the QMEAN scoring function closest to zero (QMEAN = –1.43) was chosen. The sequence identity of TMPRSS2 (model 06) created by ProMod3 was 33.82% and sequence similarity was 38%. If the target and template sequences show an amino acid identity over 30% and above, homology modeling is accepted as being reliable and successful (Xiang, 2006). To further verify model used in this study, a Ramachandran plot was created through the PROCHECK web-based tool to evaluate the energetically allowed regions of TMPRSS2 (Figure 2). According to plot statistics, 84.6% of residues were in most favored regions, 14.7% was in additional allowed regions, 0.3% was in generously allowed regions, and 0.3% of the residues were in disallowed regions. ERRAT web-based tool was also used to calculate the overall quality factor (OQF) of nonbonded atomic interactions of the model. According to this online web-based tool, the OQF of a high quality model should be above 91%. The OQF calculated by the ERRAT server of TMPRSS2, which was created as the homology model, was 92.92% (Figure 3).

### 3.2. RMSD values

RMSD values of atomic positions obtained as a result of computer-based interactions of monoterpenoid hydrocarbons with spike, TMPRSS2, CatB and CatL are given in Tables 1, 2, 3, 4, and 5, respectively. RMSD values obtained as a result of the interaction of monoterpenoids with the proteins in question were in the range of 0.02–1.97, 0.01–20.103, 0.00–13.569, and 0.01–1.14 Å, respectively. The compound showing the lowest RMSD value in interaction with spike, TMPRSS2 and CatB was camphene (0.02, 0.01, and 0.00 Å, respectively). On the other hand, RMSD values of borneol, sabinene, β-pinene, camphor, and carvone were also found to be quite low. With some exceptions, the RMSD values of monoterpenoids on all proteins were below 2.0 Å. The RMSD values obtained as a result of the interactions of menthol with TMPRSS2 and *p*-cymene with Cat B were found to be quite high (20.103 and 13.569 Å, respectively).

**Table 2:** Molecular interactions between the monoterpenoids and RBD of the spike protein of 2019-nCoV.

No	Compound	RMSD (Å)1	Binding energy (kcal/mol)	Predicted IC50 value (mM)	Classical H-bond	Van der Waals	NonclassicalH-bond(C-H, Pi-Donor)	Hydrophobic interaction		Electrostatic	Miscellaneous (Lone pairs)
								p-pinteraction	Mixedp/Alkyl		
1	Carvone	0.15	-4.70	0.360	Asn5012	Ser4942, Gly496, Phe497	-	Arg403, Tyr453, Tyr495, Tyr5052	-	-	-
2	Camphene	0.02	-4.77	0.320	-	Ser4942, Gly496, Asn5012	-	Arg403, Tyr453, Tyr495, Phe497, Tyr5052	-	-	-
3	Sabinene	0.03	-4.54	0.469	-	Ser4942, Gly496, Asn5012	-	Arg403, Tyr453, Tyr495, Phe497, Tyr5052	-	-	-
4	β-Pinene	0.12	-4.54	0.469	-	Glu406, Ser4942,Gly496, Asn5012	-	Arg403, Tyr453, Tyr495, Phe497, Tyr5052	-	-	-
5	Myrcene	0.87	-3.85	1.510	-	Glu406, Ser4942,Gly496, Asn5012	-	Tyr453, Tyr495, Arg403, Phe497,Tyr5052	-	-	-
6	α-Terpineol	0.08	-4.61	0.417	Gly496, Ser4942	Arg403, Glu406	-	Tyr453, Tyr495, Tyr5052	-	-	-
7	Thymol	0.21	-4.69	0.363	Gly496, Ser4942	Arg403, Asn5012, Tyr5052	-	Phe497, Tyr495	Tyr453	-	-
8	Carvacrol	0.05	-4.56	0.457	Tyr5052	Arg403, Ser4942, Gly496, Phe497, Asn5012	-	Tyr5052	Tyr453, Tyr495, Tyr5052	-	-
9	Camphor	1.88	-4.28	0.727	Gly496	Arg403	-	-	Tyr453,Tyr495, Tyr5052	-	-
10	β-Phellandrene	0.37	-4.56	0.453	-	Arg403, Ser4942,Gly496, Phe497, Asn5012	-	-	Tyr453, Tyr495, Tyr5052	-	-
No	Compound	RMSD (Å)1	Binding energy (kcal/mol)	Predicted IC50 value (mM)	Classical H-bond	Van der Waals	NonclassicalH-bond(C-H, Pi-Donor)	Hydrophobic interaction		Electrostatic	Miscellaneous (Lone pairs)
								p-pinteraction	Mixedp/Alkyl		
12	p-Cymene	1.97	-4.17	0.875	-	Arg403, Ser4942,Gly496, Phe497, Asn5012	-	Tyr5052	Tyr453, Tyr495, Tyr5052	-	-
13	Limonene	0.03	-4.59	0.433	-	Gln4932, Ser4942,Gly496, Asn5012	-	-	Arg403,Tyr453, Tyr495, Phe497,Tyr5052	-	-
14	Linalool	1.70	-3.60	2.290	-	Glu406, Ile418, Gly496, Asn5012	Arg403	-	Tyr453,Lys417,Arg403,Tyr453,Tyr495,Phe497,Tyr5052	-	-
15	Borneol	0.02	-4.49	0.507	Gly496, Ser4942	Arg403	-	-	Tyr453,Tyr495, Tyr5052	-	-

**Table 3:** Molecular interactions between the monoterpenoids and TMPRSS2.

No	Compound	RMSD (Å)1	Binding energy (kcal/mol)	Predicted IC50 value (mM)	Classical H-bond	Van der Waals	Nonclassical H-bond(C-H, Pi-Donor)	Hydrophobic interaction		Electrostatic	Miscellaneous (Lone pairs)
								p-pinteraction	Mixedp/Alkyl		
1	Carvone	0.07	-4.45	0.547	His279	Cys297, Thr393,Gln438, Gly439,Ser4412	Val280	-	Val280, Cys281,His279, His2962	-	-
2	Camphene	0.01	-3.79	1.660	-	Cys297, Glu299,Trp308	-	-	Pro301, Leu302,Val280, His2962	-	-
3	Sabinene	1.42	-3.48	2.800	-	His279, Cys281, His2962, Thr393, Cys437, Gln438, Gly439, Ser4412	-	-	Val280	-	-
4	β-Pinene	0.04	-3.84	1.520	-	Ser436, Cys437, Gln438, Ser4412, Ser460, Trp461, Gly462, Gly464	-	-	His2962	-	-
5	Myrcene	0.80	-3.27	3.990	-	Trp384, Thr393, Gln438, Gly439, Ser4412	-	-	Val280, Cys281, Cys297, His279, His2962	-	-
6	α-Terpineol	1.83	-4.17	0.882	Glu299	Asp338, Ser339, Thr341	-	-	Tyr337, Lys342, Lys340	-	-
7	Thymol	1.04	-3.84	1.540	His2962	Ala295, Cys297, Val298, Glu299	-	-	Val280, Pro301, Leu302	-	-
8	Carvacrol	0.57	-3.52	2.650	His279	Val278, Cys281, His2962, Thr393, Gln438, Gly439, Ser4412	-	-	Val280, His279	Val280	-
9	Camphor	0.04	-4.27	0.737	Lys340	Thr341	-	-	Lys342, Leu419, Trp461	-	-
No	Compound	RMSD (Å)1	Binding energy (kcal/mol)	Predicted IC50 value (mM)	Classical H-bond	Van der Waals	Nonclassical H-bond(C-H, Pi-Donor)	Hydrophobic interaction		Electrostatic	Miscellaneous (Lone pairs)
								p-pinteraction	Mixedp/Alkyl		
12	p-Cymene	0.86	-3.29	3.880	-	Val278, Cys281, His2962, Thr393, Gln438, Gly439, Ser4412	-	-	Val280	His279, Val280	-
13	Limonene	0.16	-3.59	2.330	-	His279, Cys297, Thr393, Gln438, Gly439, Ser4412	-	-	Val280, Cys281, His2962	-	-
14	Linalool	1.71	-2.38	17.890	Glu299	Asp338, Ser339, Lys340, Thr341	-	-	Lys342, Tyr337	-	-
15	Borneol	0.09	-4.02	1.120	Gly439, Ser4412	Gly282, Cys437, Gln438, Asp440	-	-	Val280, Cys281, His2962	-	-

**Table 4:** Molecular interactions between the monoterpenoids and CatB.

No	Compound	RMSD (Å)1	Binding energy (kcal/mol)	Predicted IC50 value (mM)	Classical H-bond	Van der Waals	NonclassicalH-bond(C-H, Pi-Donor)	Hydrophobic interaction		Electrostatic	Miscellaneous (Lone pairs)
								p-pinteraction	Mixedp/Alkyl		
1	Carvone	0.01	-5.10	0.182	His1102, His1112	Gln232, Cys262, Gly272, Gly121, Glu122	-	-	Cys119, Cys292, His1112, His1992, Trp2212	-	-
2	Camphene	0.00	-4.67	0.379	-	Gln232, Gly242, Ser25, Cys262, Gly272, Cys108, Gly121, Glu122	-	-	Cys119, His1102, His1112, Trp2212	-	-
3	Sabinene	0.08	-4.33	0.664	-	Gln232, Gly242, Ser25, Cys262, Gly272, Cys108, Cys119, Gly121, Glu122	-	-	His1102, His1112, His1992, Trp2212	-	-
4	β-Pinene	0.21	-4.56	0.456	-	Gln232, Gly242, Ser25, Cys262, Gly272, His1112, Gly121, Glu122	-	-	Cys108, Cys119, Cys292, His1102, His1992, Trp2212	-	-
5	Myrcene	1.27	-3.78	1.700	-	Gln232, Gly242, Ser25, Cys262, Gly272, Gly121	-	-	Cys108, Cys119, Cys292, His1102, His1112, His1992, Trp2212	-	-
6	α-Terpineol	1.00	-4.73	0.339	Gly242, Gly121	Gln232, Ser25, Cys262, Gly272, Cys108, Thr120, Glu122, Trp2212	-	-	Cys292, Cys119, His1102, His1112, His1992	-	-
7	Thymol	0.32	-4.20	0.836	Cys292, Gly198	Gln232, Cys262, Gly272, Gly197, Trp2212	-	His1992	Val176, Met196, Cys292, His1992	-	Cys292
No	Compound	RMSD (Å)1	Binding energy (kcal/mol)	Predicted IC50 value (mM)	Classical H-bond	Van der Waals	NonclassicalH-bond(C-H, Pi-Donor)	Hydrophobic interaction		Electrostatic	Miscellaneous (Lone pairs)
								p-pinteraction	Mixedp/Alkyl		
11	Menthol	0.05	-4.71	0.355	Glu122	Gln232, Gly242, Ser25, Cys262, Gly272, Asn72, Cys108, His1102, Gly121, Trp2212	-	-	Cys292, Cys119, His1992	-	-
12	p-Cymene	13.569	-3.93	1.320	-	Gln232, Gly242, Ser25, Gly272, His1112, Gly121, Glu122, Trp2212	-	-	Cys292, Cys108, Cys119, His1102, His1992	Cys262	Cys292
13	Limonene	0.88	-4.26	0.754	-	Gln232, Gly242, Ser25, Cys262, Gly272, Gly121,Glu122	-	-	Cys292, Cys119, Cys108, His1102, His1112, His1992, Trp2212	-	-
14	Linalool	1.15	-3.91	1.370	His1112	Gln232, Gly242, Ser25, Cys262, Gly272, Glu109,Gly121	-	-	Cys292, Cys108, Cys119, His1102, His1112, His1992, Trp2212	-	-
15	Borneol	0.14	-4.94	0.240	Gly242, Cys262, Gly121	Gln232, Ser25, Gly272, Thr120, Glu122	-	-	Cys119, His1102, His1112, Trp2212	-	-

**Table 5:** Molecular interactions between the monoterpenoids and CatL.

No	Compound	RMSD (Å)1	Binding energy (kcal/mol)	PredictedIC50 value(mM)	Classical H-bond	Van der Waals	NonclassicalH-bond(C-H, Pi-Donor)	Hydrophobic interaction		Electrostatic	Miscellaneous (Lone pairs)
								p-pinteraction	Mixedp/Alkyl		
1	Carvone	0.08	-5.03	0.205	Cys252, Trp262	Gly232, Asn662, Gly682, Val134, Asp1622, Gly164	Trp262, Gly672	-	Ala1352, Ala214, Leu692, Met1612, Met702	-	-
2	Camphene	0.12	-3.95	1.280	-	Trp262, Asn662, Gly672, Gly682, Met1612, Asp1622, Gly164	-	-	Cys252, Leu692	-	-
3	Sabinene	0.02	-4.29	0.712	-	Trp262, Gly672, Gly682, Met702, Val134, Ala1352, Met1612, Asp1622, Gly164, Ala214	-	-	Cys252, Leu692	-	-
4	β-Pinene	0.03	-3.91	1.360	-	Glu63, Asn662, Gly672,Gly682, Leu692, Met1612, Asp1622, Gly164	-	-	Cys252, Trp262	-	-
5	Myrcene	0.26	-3.92	1.340	-	Gly232, Trp262, Asn662, Gly672, Gly682, Val134, Gly164	-	-	Cys252, Ala1352, Ala214, Leu692, Met702, Cys252	-	-
6	α-Terpineol	1.09	-4.96	0.233	Gly682	Asn662, Gly672, Val134, Met1612, Asp1622, Gly164	-	-	Cys252, Leu692, Ala1352, Ala214, Met702, Cys252, Trp262	-	-
7	Thymol	0.95	-4.25	0.761	Cys252, Trp262	Asn662, Gly672, Val134, Met1612, Gly164	Trp262	-	Ala1352, Ala214, Leu692, Met702, Cys252, Leu692	Gly682, Asp1622	-
No	Compound	RMSD (Å)1	Binding energy (kcal/mol)	PredictedIC50 value(mM)	Classical H-bond	Van der Waals	NonclassicalH-bond(C-H, Pi-Donor)	Hydrophobic interaction		Electrostatic	Miscellaneous (Lone pairs)
								p-pinteraction	Mixedp/Alkyl		
9	Camphor	0.06	-3.93	1.320	Ser213	Asp71, Asp114, Lys117, Ala215, Ser216	Ala214	-	Ala214, Leu692, Met702	-	-
10	β-Phellandrene	0.08	-4.80	0.302	-	Trp262, Asn662, Gly672, Gly682, Val134, Met1612, Asp1622, Gly164	-	-	Cys252, Leu692, Ala1352, Ala214, Met702	-	-
11	Menthol	0.06	-4.80	0.303	Trp262	Cys252, Gly672, Gly682, Val134, Met1612, Asp1622, Gly164	-	-	Leu692, Ala1352, Ala214, Met702	-	-
12	p-Cymene	0.09	-4.36	0.632	-	Trp262, Asn662, Gly672, Val134, Met1612, Gly164	-	-	Ala1352, Ala214, Leu692, Met702, Cys252, Ala1352	Gly682, Asp1622	-
13	Limonene	0.05	-4.62	0.411	-	Asn662, Gly672, Gly682, Val134, Met1612, Asp1622, Gly164	-	-	Cys252, Leu692, Ala1352, Ala214, Met702, Trp262	-	-
14	Linalool	1.14	-3.80	1.650	Gly682	Asn662, Gly672, Val134, Met1612, Asp1622, Gly164	-	-	Ala1352, Ala214, Cys252, Leu692, Met702, Trp262	-	-
15	Borneol	0.01	-4.02	1.130	Asp114	Asp71, Lys117, Ser213, Ala215, Ser216	-	-	Ala214, Leu692, Met702	-	-

### 3.3. Binding energies

The binding energies of monoterpenoids to target molecules ranged from –5.01 to –2.38 kcal/mol. The binding energies between monoterpenoids and spike, TMPRSS2, CatB and CatL were in the ranges of –5.0/–3.6, –4.45/–2.38, –5.53/–3.78, and –5.03/–3.8 kcal/mol, respectively. The binding energy of carvone against all proteins was found to be significantly lower than the others. In addition, the binding energies of menthol, camphor, and α-terpineol were also found to be promising. Linalool and myrcene were the molecules with the highest binding energy against all proteins.

### 3.4. Predicted IC50 values

The predicted IC50 values of monoterpenoids against spike, TMPRSS2, CatB, and CatL were in the range of 0.213–2.29, 0.547–17.89, 0.087–1.7 and 0.205–1.65 mM, respectively. As with the binding energies of the molecules, the IC50 value of carvone was found to be significantly lower than other monoterpenoids. In addition, the IC50 values of camphor, menthol, and α-terpineol against all protein targets were also low. IC50 values of myrcene and linalool were quite high compared to other monoterpenoids. In particular, IC50 value of linalool against TMPRSS2 was determined as 17.89 mM.

### 3.5. Bonding interactions

The bonds between the monoterpenoids and contact residues in the active regions of the target proteins are detailed in Tables 1, 2, 3, and 4. Van der Waals and hydrophobic interactions (both p-p and mixed p/alkyl) played an important role in the interaction between monoterpenoids and RBD of the spike protein of 2019-nCoV. Van der Waals forces and hydrophobic interactions also played a decisive role in the molecular interactions between monoterpenoids and TMPRSS2, CatB, and CatL. However, it was found that hydrophobic effects between monoterpenoids and target proteins were rather established through mixed p/alkyl interactions.

**Figure 4:**
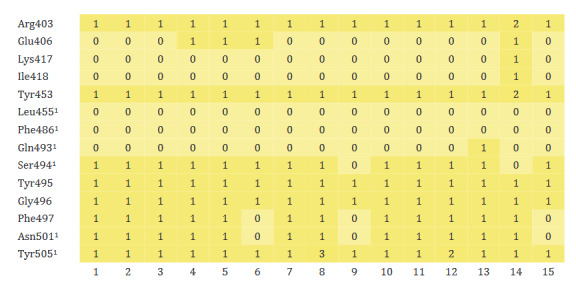
Heatmap of monoterpenoid/RBD of the spike protein of 2019-nCoV interaction. ^1^Amino acid residues involved in binding to ACE2 in the receptor binding domain of 2019-nCoV (1: Carvone, 2: Camphene, 3: Sabinene, 4: β-Pinene, 5: Myrcene, 6: α-Terpineol, 7: Thymol, 8: Carvacrol, 9: Camphor, 10: β-Phellandrene, 11: Menthol, 12: p-Cymene, 13: Limonene, 14: Linalool, 15: Borneol).

In general, no interaction has been formed between monoterpenoids and Leu455, Phe486, and Gln493, amino acids involved in binding ACE2 in the RBD of the spike protein of 2019-nCoV (Figure 4). However, it was found that molecular interaction was formed between other active amino acids (Asn501 and Tyr505) and monoterpenoids. In addition, monoterpenoids were found to interact with other amino acids (Arg403, Tyr453, Tyr495, Gly496 and Phe497) of the spike protein.

**Figure 5:**
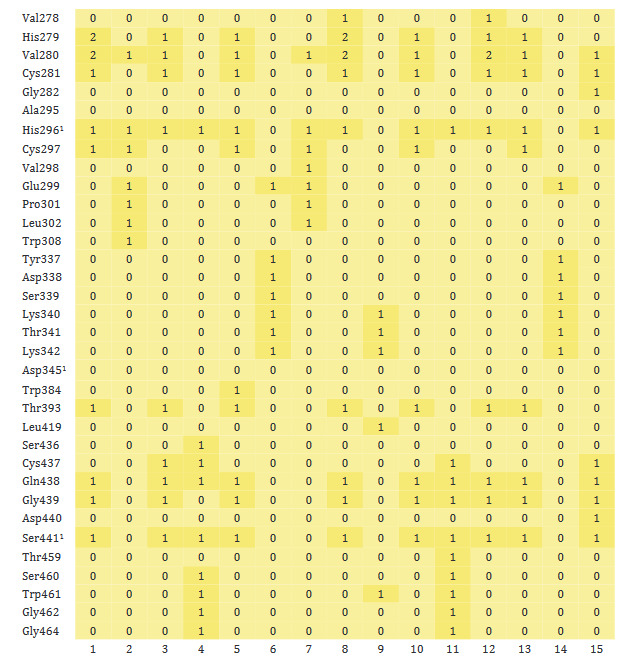
Heatmap of monoterpenoid/TMPRSS2 interaction. ^1^ The active amino acid residues of TMPRSS2 (1: Carvone, 2: Camphene, 3: Sabinene, 4: β-Pinene, 5: Myrcene, 6: α-Terpineol, 7: Thymol, 8: Carvacrol, 9: Camphor, 10: β-Phellandrene, 11: Menthol, 12: p-Cymene, 13: Limonene, 14: Linalool, 15: Borneol).

As understood from heatmap, which shows the molecular interaction between monoterpenoids and TMPRSS2 (Figure 5), carvone and carvacrol were the most intensely interacting molecules with the protein in question. On the other hand, the number of bonds between TMPRSS2 and camphene, α-terpineol, thymol, camphor, and linalool was less than others. While molecular interaction was formed between monoterpenoids and the active amino acids of TMPRSS2, His296 and Ser441, no interaction with the other active amino acid, Asp345, was achieved. Monoterpenoids also showed several interactions with some other amino acids of TMPRSS2 (His279, Val280, Cys281, Thr393, Gln438, and Gly439).

**Figure 6:**
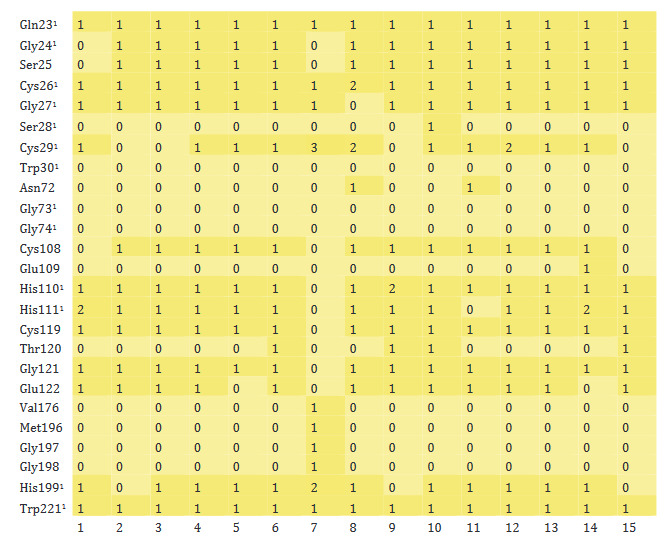
Heatmap of monoterpenoid/CatB interaction. ^1^ The active amino acid residues of CatB (1: Carvone, 2: Camphene, 3: Sabinene, 4: β-Pinene, 5: Myrcene, 6: α-Terpineol, 7: Thymol, 8: Carvacrol, 9: Camphor, 10: β-Phellandrene, 11: Menthol, 12: p-Cymene, 13: Limonene, 14: Linalool, 15: Borneol).

The vast majority of the active amino acids of CatB (Gln23, Gly24, Cys26, Gly27, Cys29, His110, His111, His199 and Trp221) were found to have formed interactions with monoterpenoids (Figure 6). However, there was no interaction occurred between some active amino acids (Ser28, Trp30, Gly73, and Gly74) and phytochemicals. Intermolecular bonds were also formed between monoterpenoids and other amino acids of CatB (Ser25, Cys108, Cys119, Gly121, and Glu122). The monoterpenoids that interacted most with the amino acids of CatB were β-phellandrene, α-terpineol, *p*-cymene, and linalool.

**Figure 7:**
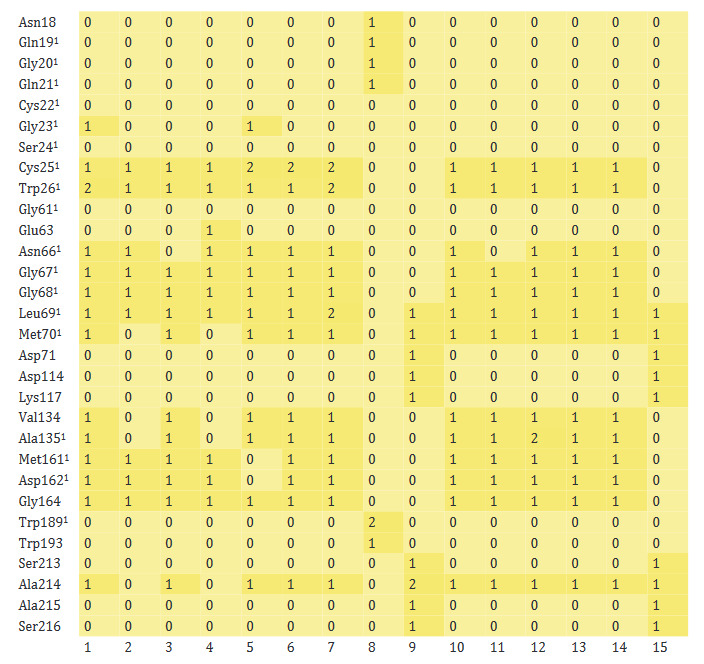
Heatmap of monoterpenoid/CatL interaction. ^1^The active amino acid residues of CatL (1: Carvone, 2: Camphene, 3: Sabinene, 4: β-Pinene, 5: Myrcene, 6: α-Terpineol, 7: Thymol, 8: Carvacrol, 9: Camphor, 10: β-Phellandrene, 11: Menthol, 12: p-Cymene, 13: Limonene, 14: Linalool, 15: Borneol).

The heatmap showing the interaction of monoterpenoids with CatL is given in Figure 7. Monoterpenoids formed several bonds with about 60% of the active amino acids of CatL (Cys25, Trp26, Asn66, Gly67, Gly68, Leu69, Met70, Ala135, Met161, and Asp162). However, the monoterpenoids could not form any interactions with other active amino acids (Gln19, Gly20, Gln21, Cys21, Gly23, Ser24, Gly61, and Trp189) or the interaction was rather weak. Monoterpenoids also formed bonds with Val134, Gly164 and Ala214, which are nonactive residues of CatL. The number of bonds established between CatL and thymol, carvone, α-terpineol and *p*-cymene was found to be higher than other monoterpenoids.

### 3.6. RBCI values of monoterpenoids

**Figure 8:**
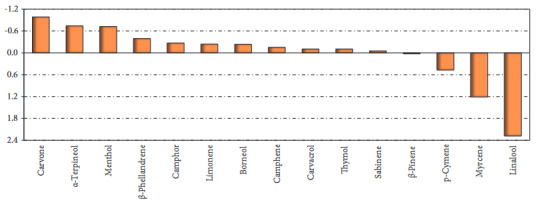
RBCI values of monoterpenoids.

As known, in this study, the molecular interaction between 15 different monoterpenoids and 4 different protein targets was analyzed. Binding energy and IC50 data were obtained in the interaction analysis for each protein-ligand system. In order to detect ‘hit’ monoterpenes, a new analysis method called RBCI, the details of which are given in the ‘Material and methods’ section, has been applied. In order to calculate the RBCI values of phytochemicals, the binding energy and IC50 values of each monoterpene against all proteins were used together. It is not possible to use all data sets, each of which has different scientific meanings, in order to rank about the effectiveness of phytochemicals on target proteins. However, with RBCI analysis, the effectiveness of phytochemicals on all proteins can be determined by using different data sets at the same time. Thus, the most effective protein on all 4 proteins can be documented statistically (Figure 8).

**Figure 9:**
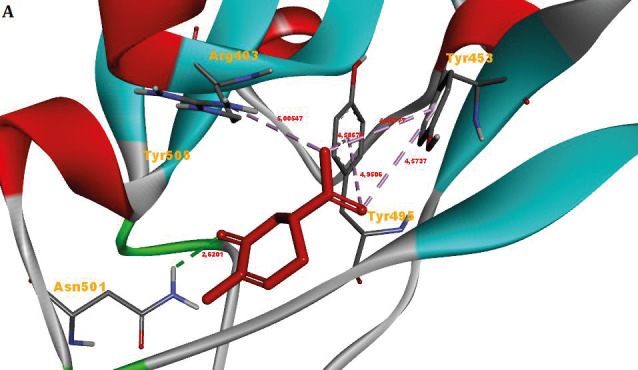

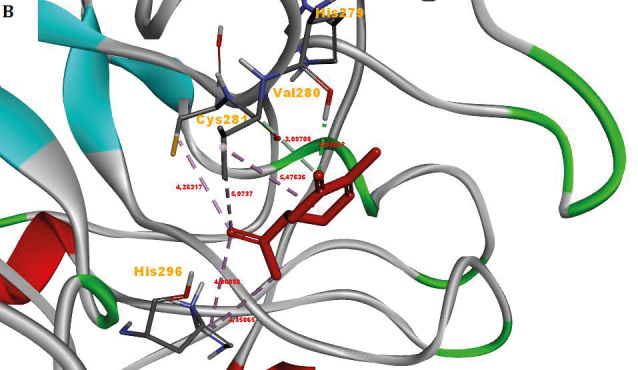

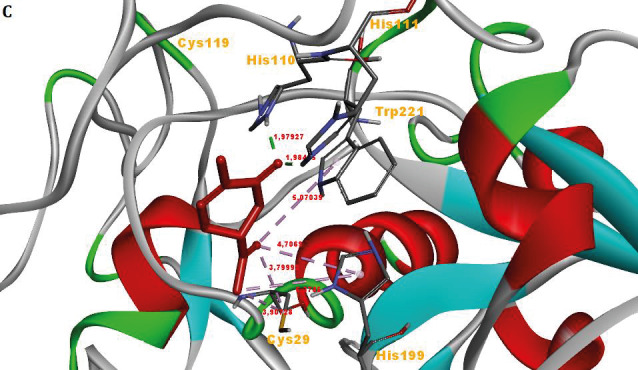

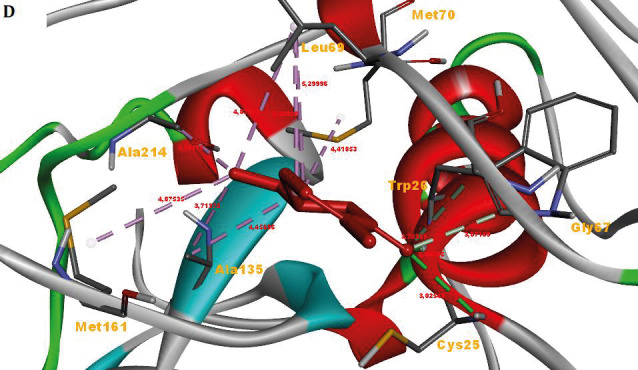
Top ranked conformation of carvone in the A) RBD of the spike protein of 2019-nCoV, B) TMPRSS2, C) CatB, and D) CatL.

RBCI analysis resulted in the superiority of carvone. Top ranked conformation of carvone in the RBD of the spike protein of 2019-nCoV, TMPRSS2, CatB, and CatL are presented in Figure 9. RBCI analysis also confirmed that *p*-cymene, myrcene, and linalool are the weakest components against protein targets in question.

### 3.7. Pharmacokinetic properties of monoterpenoids

Drug-likeness properties of docked monoterpene hydrocarbons against spike glycoprotein of 2019-nCoV, TMPRSS2, CatB and CatL and their ADMET profiles are given in Tables 6 and 7, respectively. Carvone, myrcene, α-terpineol, thymol, carvacrol, β-phellandrene, α-terpinene, limonene, linalool, and borneol were determined to be molecules in accordance with Lipinski’s rule-of-five (Lipinski et al., 1997). However, the Moriguchi Log P (MLOGP) values of camphene, sabinene, β-pinene, α-thujene, and *p*-cymene have been found to be higher than 4.15. It has been determined that all molecules can cross the blood-brain barrier (BBB). None of the molecules were substrates of P-glycoprotein (P-gp). While camphene, β-pinene, thymol, carvacrol, *p*-cymene, and limonene inhibited CYP2C9, CYP1A2, and CYP2D6, other monoterpenoids had no inhibitory effect on cytochromes. None of the monoterpenoids exhibited toxic effect. The LD50 doses of the molecules in rats were in the range of 1.549–2.074 mol/kg. While thymol and carvacrol were monoterpenoids with the highest LD50 value, the LD50 values of sabinene and camphene were found to be lower than others.

**Table 6:** Drug-likeness properties of docked monoterpene hydrocarbons against spike glycoprotein of 2019-nCoV, TMPRSS2, CatB and CatL.

No	Compound	Number of rotatable bonds	TPSA1	Consensus Log P	Log S(ESOL2)	Drug-likeness(Lipinski’s rule-of-five)
1	Carvone	1	17.07 Å2	2.44	-2.41	Yes (0 Violation)
2	Camphene	0	0.00 Å2	3.43	-3.34	Yes (1 violation; MLOGP3> 4.15)
3	Sabinene	1	0.00 Å2	3.25	-2.57	Yes (1 violation; MLOGP > 4.15)
4	β-Pinene	0	0.00 Å2	3.42	-3.31	Yes (1 violation; MLOGP > 4.15)
5	Myrcene	4	0.00 Å2	3.43	-3.05	Yes (0 Violation)
6	α-Terpineol	1	20.23 Å2	2.58	-2.87	Yes (0 Violation)
7	Thymol	1	20.23 Å2	2.80	-3.19	Yes (0 Violation)
8	Carvacrol	1	20.23 Å2	2.82	-3.31	Yes (0 Violation)
9	α-Thujene	1	0.00 Å2	3.15	-2.41	Yes (1 violation; MLOGP > 4.15)
10	β-Phellandrene	1	0.00 Å2	3.07	-2.79	Yes (0 Violation)
11	α-Terpinene	1	0.00 Å2	3.30	-3.30	Yes (0 Violation)
12	p-Cymene	1	0.00 Å2	3.50	-3.63	Yes (1 violation; MLOGP > 4.15)
13	Limonene	1	0.00 Å2	3.37	-3.50	Yes (0 Violation)
14	Linalool	4	20.23 Å2	2.66	-2.40	Yes (0 Violation)
15	Borneol	0	20.23 Å2	2.38	-2.51	Yes (0 Violation)

**Table 7:** ADMET profiles of monoterpene hydrocarbons.

No	Compound	BBB1 permeation	P-gp substrate2	CYP3 inhibition	AMES toxicity	Hepatotoxicity	LD50 in rat(mol/kg)
1	Carvone	Yes	No	No	No	No	1.707
2	Camphene	Yes	No	Yes (CYP2C9)	No	No	1.554
3	Sabinene	Yes	No	No	No	No	1.549
4	β-Pinene	Yes	No	Yes (CYP2C9)	No	No	1.673
5	Myrcene	Yes	No	No	No	No	1.643
6	α-Terpineol	Yes	No	No	No	No	1.923
7	Thymol	Yes	No	Yes (CYP1A2)	No	Yes	2.074
8	Carvacrol	Yes	No	Yes (CYP1A2)	No	Yes	2.074
9	α-Thujene	Yes	No	No	No	No	1.589
10	β-Phellandrene	Yes	No	No	No	No	1.709
11	α-Terpinene	Yes	No	No	No	No	1.766
12	p-Cymene	Yes	No	Yes (CYP2D6)	No	No	1.827
13	Limonene	Yes	No	Yes (CYP2C9)	No	No	1.880
14	Linalool	Yes	No	No	No	No	1.704
15	Borneol	Yes	No	No	No	No	1.707

## 4. Discussion

RMSD is a value commonly used in molecular docking analysis to measure the reproduction quality of a known binding pose of the molecule couples. This value is functional when the ligand molecule shows different poses in the binding site of the protein. Researchers suggest that RMSD values less than 2.0 Å are an important indicator of the quality of binding poses (Ramírez and Caballero, 2018). Accordingly, almost all of the analyzed monoterpenoids were found to have RMSD values below 2.0 Å against protein targets. It was found that RMSD values obtained only from menthol-TMPRSS2 and *p*-cymene-CatB interactions were above this critical limit and therefore could not exhibit sufficient binding activity on these molecules.

There was no correlation between the RMSD values of the molecules and binding energies and predicted IC50 values. However, a high correlation was found between binding energies obtained against all protein targets and IC50 values.

As a result of docking analysis, monoterpenoids interacted with more than half of the active amino acids of protein targets. Monoterpenoids mostly interacted with the active amino acids of CatB and TMPRSS2 (69% and 66% of active amino acids, respectively). Monoterpenoids bound with 55% of active amino acids of CatL, while this ratio has decreased to 50% in spike glycoprotein.

**Figure 10:**
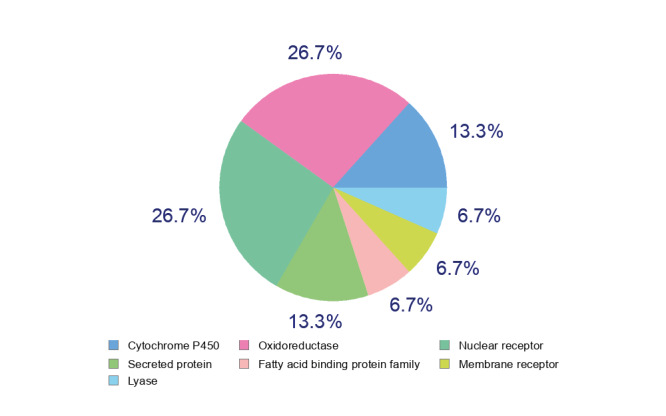
Target prediction of carvone.

As a result of the RBCI analysis performed to detect the most effective phytochemicals against the all protein targets, carvone was clearly superior to others. It was found to exhibit drug-like property according to the Lipinski’s rule-of-five. As it is understood from the ADMET profile, carvone can effectively pass through BBB. P-gp is an important cell membrane component responsible for the removal of toxic molecules from the cell. This protein, which is found in many organisms, is thought to be one of the cell’s defense mechanisms against harmful substances (Nguyen et al., 2020). As can be seen from Table 7, where ADMET profiles of monoterpenoids were given, carvone is not a substrate for P-gp. This finding shows that carvone is not perceived by the cell as a toxic molecule. In addition, the results of AMES toxicity and hepatotoxicity analysis support this finding. Carvone has also been found not to inhibit CYPs involved in the oxidation of xenobiotics and some endogenous harmful compounds (Rettie and Jones, 2005; Spector and Kim, 2015). This finding means that with carvone intake, metabolic activities based on oxidation can continue without disruption. LD50 dose of carvone in rats was also found to be better than those of sabinene, camphene, α-thujene, myrcene, β-pinene, and linalool. In Figure 10, the intracellular targets of carvone are included. According to this pie chart, the vast majority of intracellular targets of carvone are oxidoreductases and nuclear receptors. Oxidoreductases are enzymes that transfer the electron from the donor molecule to the acceptor. Usually they use NADP or NAD+ as the cofactors. In many organisms, transmembrane oxidoreductases are known to form the electron transfer chain (Rac and Fulgosi, 2020). Nuclear receptors are responsible for delivering the signal carried by some hormones (e.g., thyroid or some steroidal hormones) to the nucleus of the cell. Thus, these proteins regulate the expression of certain specific genes along with some other proteins (Evans, 1988). Although it is known that carvone targets these proteins, it is thought that detailed analyzes should be made to understand whether carvone has an inhibitory and/or activator effect on the activities of these proteins.

As a result of the literature research, the molecular interaction between spike glycoprotein of 2019-nCoV and carvone, camphene, α-terpineol, thymol, carvacrol, camphor, β-phellandrene, menthol, linalool, and borneol was analyzed and the binding energies of these molecules were found to be in the range of –3.4/–3.7 kcal (Smith and Smith, 2020). The data in question were found to be weaker than the data presented in the current study. However, no reports of the interaction of other monoterpenoids with the spike protein have been found. Additionally, no study was found documenting the interaction between neither carvone nor other monoterpenoids and TMPRSS2, CatB, and CatL. Therefore, the data presented here were considered to be the first records for the literature.
